# Effect of phytate on crystallization on ureteral stents and bacterial attachment: an in vitro study

**DOI:** 10.1007/s00240-022-01350-1

**Published:** 2022-09-05

**Authors:** Paula Calvó, Margalida Mateu-Borras, Antonia Costa-Bauza, Sebastián Albertí, Fèlix Grases

**Affiliations:** 1 Laboratory of Renal Lithiasis Research, University Institute of Health Sciences Research (IUNICS-IdISBa), University of Balearic Islands, 07122 Palma, Spain; 2 Laboratory of Infection and Immunity, University Institute of Health Sciences Research (IUNICS-IdISBa), University of Balearic Islands, 07122 Palma, Spain

**Keywords:** Bacteria, Phytate, Catheter, Crystallization, Calcium phosphate, Deposits

## Abstract

The use of double J ureteral stents can lead to several adverse effects, as urinary infection. Bacteria tend to colonize the stent surface, leading to the formation of bacterial biofilms. The presence of urease-producing bacteria increase the urine pH leading to the incrustation and blockage of the stent. On the other hand, these large crystalline masses function as niduses, allowing the attachment of even more bacteria and decreasing its exposure to antibiotics. The aim of this in vitro study was to assess the effect of phytate on the attachment of bacteria to the catheter surface under conditions that favor crystallization. Catheter sections were incubated in a synthetic urine medium (pH 6.5) in the presence or absence of *Pseudomonas aeruginosa* and phytate. Amount of calcium deposits was measured using an Arsenazo III colorimetric method and the number of attached bacteria to the stent was determined. Differences were assessed using an ANOVA with a Bonferroni post hoc test. The formation of calcium phosphate deposits (brushite and hydroxyapatite) and oxalate crystals (COM), as were as the amount of bacteria decreased when phytate was present. Thus, phytate successfully decreased bacterial adhesion by inhibiting the formation of crystalline deposits.

## Introduction

A ureteral stent is an important medical device that can restore the normal flow of urine from the kidney to the bladder when there is an obstruction. These stents promote lumen dilatation and restore normal flow [1–3]. Although they typically resolve the problem of obstruction, the introduction of a stent can lead to adverse effects, such as an infection in the urinary system, and the stent can also become encrusted and obstructed over time [2].

The risk of a urinary tract infection (UTI) is linearly related to the duration of stent indwelling, and use of these stents for 30 days or more often leads to bacteriuria. Microorganisms tend to colonize the stent surface, leading to the formation of bacterial biofilms [4]. The presence of urease producing bacteria increase the urine pH due to their hydrolysis of urea into ammonia. When the urine pH is greater than 6.2, deposits of hydroxyapatite (HAP, Ca_10_[PO_4_]_6_[OH]_2_) and struvite (NH_4_MgPO_4_·6H_2_O) can form on the stent, leading to incrustation and blockage [5–7]. Incrustation may also occur without bacterial infection, because the stent can function as a heterogeneous nucleant for substances that are supersaturated in the urine [8–10].

The duration of catheter indwelling increases the risk of stent encrustation and blockage [11–13], and can lead to a rigid catheter with reduced tensile strength [14]. This increases the risk of ureter trauma or injury during stent removal, and the leakage of the urine within the body.

The relationship between biofilm formation and catheter encrustation is unclear, although some studies of urethral and ureteral stents have shown a clear feedback during the process. On the one hand, bacterial adhesion and biofilm formation on the catheter surface can promote crystal deposition and encrustation [4, 15, 16]; on the other hand, encrustation may provide niduses for the colonization of bacteria and the formation of a bacterial crystal biofilm [17–19].

Previous research reported that consumption of myo-inositol hexaphosphate (InsP6, phytate) can inhibit the formation of renal calculi [20] and reduce the encrustation of ureteral stents [10]. The aim of the present in vitro study is to assess the effect of phytate on the attachment of bacteria and the formation of niduses on the catheter surface when conditions are favorable for the formation of calcium phosphate crystals.

## Materials and methods

### Solutions and batch system

Synthetic urine was prepared by a making fresh mixture of equal volumes of two solutions, with solution A containing CaCl_2_ and solution B containing Na_2_C_2_O_4_ (Table 1). The pH of each solution was adjusted to 6.5, a condition that promotes crystallization of calcium phosphate and calcium oxalate monohydrate (COM). Glucose and albumin (Sigma-Aldrich, St. Louis, USA) were added to solution B. Glucose was the carbon source for bacterial growth, and albumin was a mimic of the proteinaceous debris present in infected urine. Different amounts of phytic acid sodium salt (P8810, Sigma-Aldrich, St. Louis, USA) were also added to solution B. The synthetic urine components were obtained from PanReac (Barcelona, Spain).

**Table 1:** Composition of synthetic urine

Solution A		Solution B	
Na_2_SO_4_·10H_2_O	19.34 mM	NaH_2_PO_4_·2H_2_O	15.45 mM
MgSO_4_·7H_2_O	5.92 mM	Na_2_HPO_4_·12H_2_O	15.64 mM
NH_4_Cl	86.75 mM	NaCl	223.31 mM
KCl	162.69 mM	Na_2_C_2_O_4_	0.6 mM
CaCl_2_	8.5 mM	Glucose	20 g/L
		Albumin	2 g/L
		Phytate	0, 2.4, or 4.8 µM

**Fig. 1:**
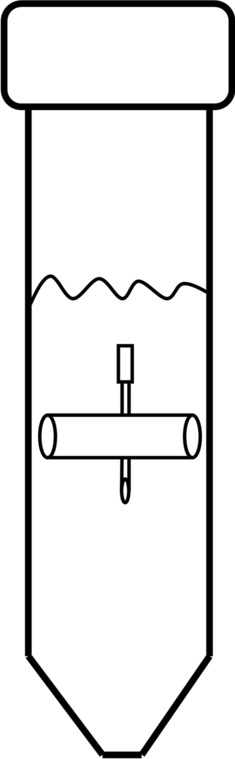
Experimental system. A 1.5 cm section of a hydro-coated silicon catheter was attached to a needle inside a 50 mL Falcon tube that was incubated with 40 mL of synthetic urine (pH 6.5) at 37 °C for 24 h

Experiments were performed in sterile 50 mL Falcon tubes. Each tube contained a hydro-coated silicon catheter section that was 1.5 cm long (BCHG64, Coloplast, Humlebaek, Denmark) and attached to a needle for support (Fig. 1). Each Falcon tube was filled with 20 mL of solution A and 20 mL of solution B, and maintained for 24 h at 37 °C.

### Calcium analysis

After 24 h, each catheter deposit was dissolved with 0.5 M HCl, and resulting solution was adjusted to pH 3.0 for analysis. Calcium was determined using an Arsenazo III colorimetric method [21]. All measurements were performed in triplicate.

### Bacterial attachment assays

Bacterial attachment assays were performed using *P. aeruginosa* reference strain PAO1, which was originally isolated from an infected wound [22]. For these assays, the strain was grown 18 h at 37 °C in artificial urine without phytic acid.

Catheter sections of 1.5 cm were placed into sterile tubes containing 40 mL of artificial urine (20 mL solution A + 20 mL solution B) without or with phytate (0, 1.2, or 2.4 μM). Tubes were inoculated with bacterial cells of P. aeruginosa PAO1 that were grown overnight in synthetic urine without phytate to a final concentration of 10^8^ viable bacterial cells/mL and incubated 24 h at 37 °C without shaking. After this incubation, catheter sections were transferred to new sterile microfuge tubes using sterile forceps and rinsed with 4 mL of sterile phosphate buffered saline (PBS) to remove unattached bacteria. Next, catheter sections were placed in new microfuge tubes containing 1 mL of PBS that were centrifuged at 13,000×*g* for 30 min to harvest the bacterial cells. Finally, the catheter sections were discarded, and the pellet of bacteria detached from the catheters was resuspended in 1 mL of PBS. Bacterial cells were quantified by plating appropriate dilutions of the bacterial suspension on Luria Bertani agar plates. Results were expressed as Colony Forming Units (CFU) per mL of PBS. A negative control, consisting in catheter without bacteria, was processed in parallel.

### Scanning electron microscopy

The morphological and structural characteristics of the deposits that formed on the surface of the stent in absence and presence of InsP6 and *P. aeruginosa* were examined using scanning electron microscopy (SEM, Hitachi S-3400N) coupled with RX energy dispersive microanalysis (Bruker AXS XFlash Detector 4010). Stents were washed with Mili-Q water to remove artificial urine residues to avoid the formation of NaCl and KCl crystals. After washing them, they were left to dry for 1 day at room temperature. Afterwards, stents were stuck on a sample holder with adhesive tape to avoid their displacement and observed with SEM without any other preparation.

### Statistics

Normality graphs were used to assess data distributions. Data were presented as means and standard errors of the means (SEMs). The amounts of calcium and attached bacteria in the different groups were compared using an ANOVA with a Bonferroni post hoc test. A two-tailed *p* value less than 0.05 was considered statistically significant. Statistical analyses were performed using SPSS version 25.0 (SPSS Inc., Chicago, IL, USA).

## Results

### Effect of phytate on formation of calcium crystals

**Fig. 2:**
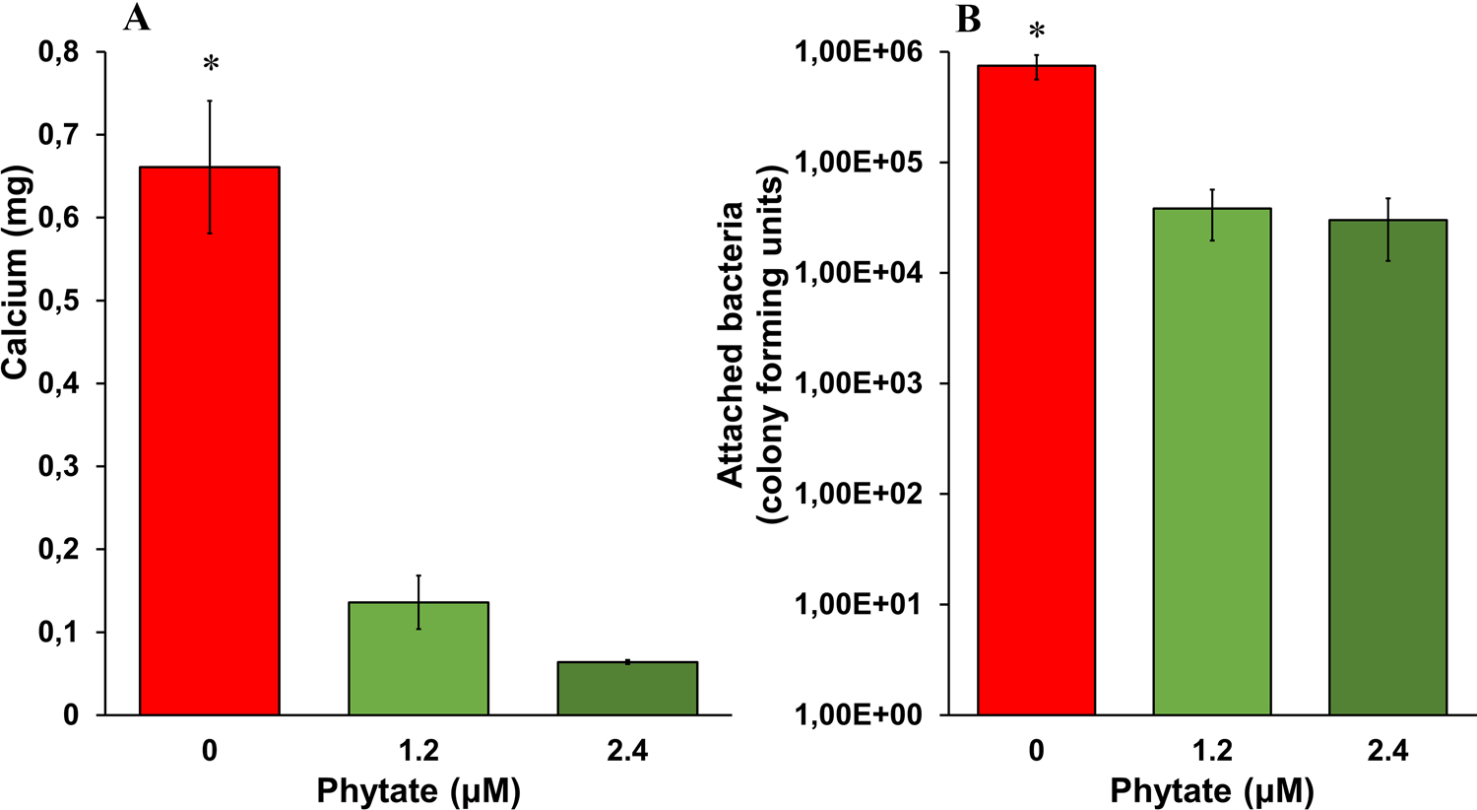
**A** Effect of phytate on the formation of calcium deposits on catheter sections. Means and SEMs are indicated. *An ANOVA and a Bonferroni post-hoc test indicated the control group (0 µM phytate) had a greater level of calcium (*p* value < 0.05) than the phytate groups, but there was no significant difference in the phytate groups. **B** Effect of phytate on the attachment of bacteria (*P. aeruginosa* PAO1) to catheter sections. Means and SEMs are indicated. *An ANOVA and a Bonferroni post-hoc test indicated the control group (0 µM phytate) had more attached bacteria (*p* value < 0.05) than the phytate groups, but there was no significant difference in the phytate groups

**Fig. 3:**
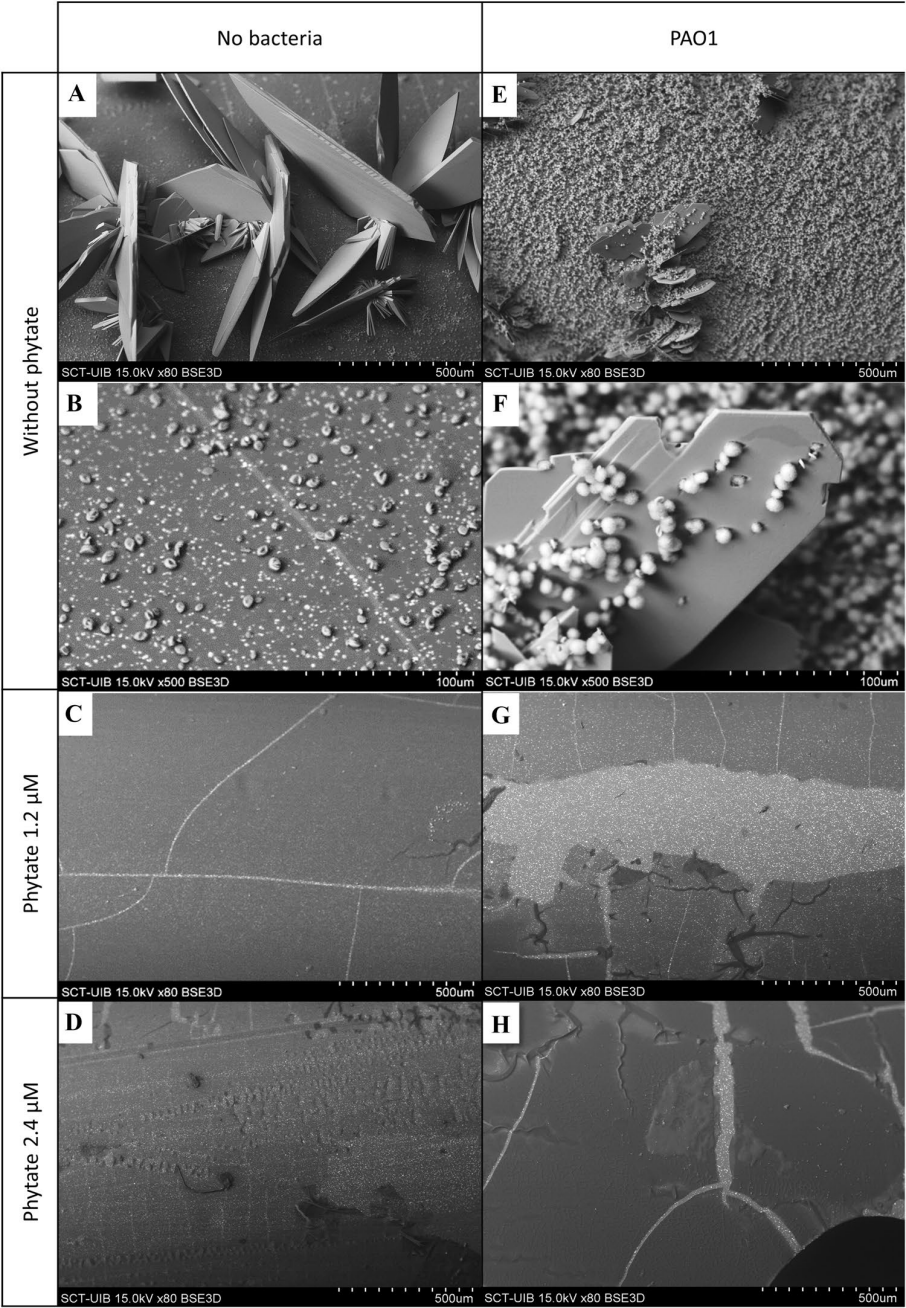
Scanning electron microscopy of catheter sections after incubation in synthetic urine with different concentrations of phytate and PAO1. The magnifications at which the photographs were taken are indicated after the “x” symbol on the black band, in which the scale bar also appears. **A** Without phytate and bacteria, brushite crystals and a thin layer of COM deposits were uniformly distributed on the catheter surface. **B** Higher magnification shows that COM deposits covered the organic matter on the catheter surface, and the catheter surface appeared as a “crack” from top-left to bottom-right. **C **When the phytate level was 1.2 µM, there were no evident crystals, but “cracks” were present on the thin layer of organic matter on the catheter surface. **D** When the phytate level was 2.4 µM, there were no evident crystals, but “cracks” were present on the thin layer of organic matter on the catheter surface due to dehydration processes. **E** Without phytate but with PAO1, there were large and uniformly distributed brushite crystals with a layer of HAP. **F** Higher magnification shows evidence of HAP coating of the brushite crystals. **G** When the phytate level was 1.2 µM, there were no evident crystals, but a layer of organic matter with “cracks” was evident. **H** When the phytate level was 2.4 µM, there were also no evident crystals, but a layer of organic matter with “cracks” was evident

We first determined the amount of calcium deposited on the catheters after 24 h of incubation with different concentrations of phytate (Fig. 2A). The results indicated that the formation of calcium phosphate deposits (brushite and hydroxyapatite) and oxalate crystals (COM) decreased when phytate was added. Scanning electron microscopy of catheter sections confirmed that phytate inhibited the formation of brushite and COM crystals. The presence of phytate led to only a thin layer of organic matter on the catheter surface (Fig. 3C, D, G and H).

### Effect of phytate on attachment of bacteria

**Fig. 4:**
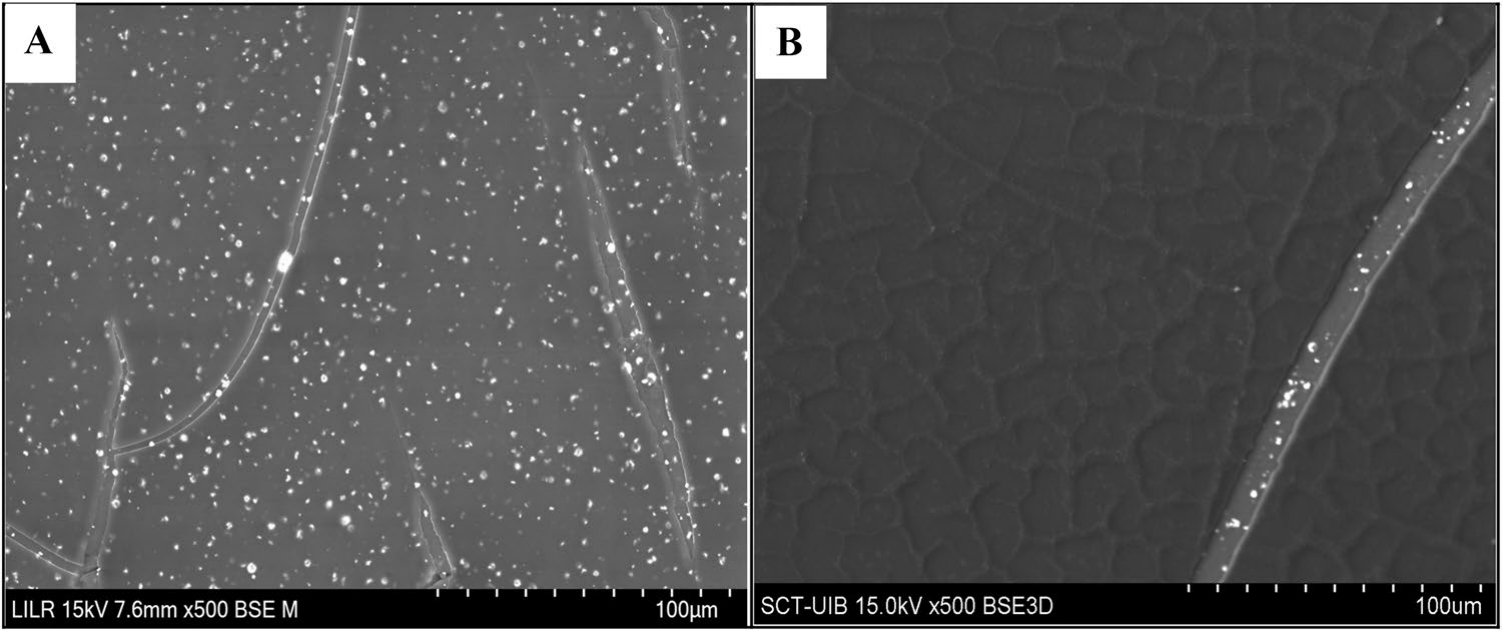
Scanning electron microscopy of a catheter section before use compared whit a section after incubation in synthetic urine. **A** Catheter section before use presents a thin broken layer that corresponds with the hydro coating of commercial catheters. **B** On the other hand, the layer formed after incubation of the catheter with artificial urine containing albumin and glucose is thicker and coats the original surface of the catheter before use

We also measured the levels of bacteria that were attached to the catheters after 24 h of incubation with different concentrations of phytate (Fig. 2B). Similar to the crystallization experiments, the amount of bacteria decreased by more than tenfold when phytate was added. Scanning electron microscopy of the catheter sections indicated that brushite, HAP, and COM crystals formed when the solution contained no phytate (Fig. 3E, F). However, only a thin layer of organic matter was detected when phytate was present in the solution (Fig. 3G, H). To confirm that the layer observed was organic matter and not only the hydro coating of the catheter, a picture of a catheter section before use was taken (Fig. 4A).

## Discussion

The formation of biofilm on a ureteral stent is a multistep process that is not yet completely understood [14]. Some researchers found that UTIs lead to the development of catheter biofilms and raise the urinary pH above 6.2. This elevated pH favors the crystallization of struvite and HAP. This crystalline layer then provides niduses for the establishment of even more bacteria.

However, a crystalline layer can also form on the catheter surface in the absence of infection [9, 10], depending on urine composition and pH. This is consistent with the observation of other researchers who found that the first step in the development of bacterial biofilms was the formation of a crystalline layer [18, 19]. This initial calcium-based crystalline layer allows bacterial attachment and biofilm formation, and these bacteria can increase the pH to above 6.2. This elevated pH then favors the formation of additional deposits, such as struvite. These large crystalline masses function as niduses, allowing the attachment of even more bacteria [17]. Thus, there appears to be a positive feedback loop of stent crystallization and bacterial colonization.

Although the details of stent crystallization and bacterial colonization are not completely elucidated, it seems important to prevent the formation of crystalline deposits to prevent bacterial proliferation, whichever process occurs first. Because a high urine pH favors the formation of calcium deposits, we performed our in vitro experiments at a pH of 6.5 to promote the formation of calcium-based crystals, such as calcium phosphate (brushite and HAP) and calcium oxalate. Notably, phytate is well-known to inhibit the formation of these types of deposits, and we, therefore, evaluated the effect of phytate on crystallization and bacterial attachment.

The results showed that phytate was effective in preventing the formation of a calcium-based crystalline layer in the absence of bacteria (Fig. 3A–D) and in the presence of *P. aeruginosa* (Fig. 3E–H). Thus, brushite, HAP and calcium oxalate deposits were minimal on stents that were incubated with 1.2 or 2.4 μM phytate. This was supported by our measurements of the level of calcium concentration (Fig. 2A), which indicated much lower levels when the stents were incubated with phytate. Moreover, the layer of organic matter that formed in the absence of bacteria (Fig. 3C, D) and in presence of bacteria (Fig. 3G, H) had a similar morphology.

A more than tenfold decrease in the number of bacteria on the catheter surface when the artificial urine included 1.2 or 2.4 μM phytate (Fig. 2B) was also observed. This finding, together with our results on crystal formation, indicated that phytate reduced the number of bacteria attached on a stent surface by preventing the formation of the crystalline layer. It is important to prevent niduses and crystalline mass formation, because this can occlude the lumen of the ureter and generate significant clinical complications, as well as persistent urinary tract infections.

Finally, crystal formation and bacterial adherence on the stent were each similar at phytate concentrations of 1.2 and 2.4 µM. At both concentrations, very few crystals formed, and the few bacteria that were present adhered directly to the surface or the interior lumen of the catheter. This confirms that bacteria adhere to a catheter surface even in the absence of a crystalline layer, forming an initial biofilm layer with the organic matter. Therefore, from the results of this research it was conclude that phytate successfully decreased the number of bacteria that adhered to a catheter surface by inhibiting the formation of a calcium-based crystalline layer, which functions as locus for bacterial colonization and growth.

Early prevention of the formation of calcium-based deposits could be a key to preventing subsequent persistent urinary system infections and their many adverse consequences. In addition, our results indicated that the large crystals on a catheter can serve as a locus for bacterial growth, because there are far fewer bacteria on the catheter surface when these crystals do not develop. It is likely that the absence of catheter deposits in vivo may also increase exposure of bacteria on the catheter surface to antibiotics.
